# Successful pregnancy and fetal outcome following previous treatment with pembrolizumab for relapsed Hodgkin's lymphoma

**DOI:** 10.1002/cnr2.1432

**Published:** 2021-05-28

**Authors:** Alexandre Le‐Nguyen, Ryan N. Rys, Tina Petrogiannis‐Haliotis, Nathalie A. Johnson

**Affiliations:** ^1^ Department of Medicine McGill University Montreal Quebec Canada; ^2^ Lady Davis Institute for Medical Research Jewish General Hospital Montreal Quebec Canada; ^3^ Department of Pathology, Jewish General Hospital McGill University Montreal Quebec Canada

**Keywords:** immunotherapy, hematological cancer, lymphoma, medical oncology

## Abstract

**Background:**

Classical Hodgkin lymphoma (cHL) is one of the most frequently diagnosed neoplasms in young adults and is curable even in the relapse setting. Many patients seek advice regarding pregnancy once they have a sustained complete remission (CR). PD1 inhibitors are effective in inducing CRs in relapsed cHL, but little is known about their effects on pregnancy, fetal outcomes, or risk of relapse. The PD1/PDL1 axis is vital in the maintenance of pregnancy, allowing for fetal tolerance. This axis is also a key pathway by which Hodgkin Reed Sternberg cells escape immune surveillance. Thus, exposure to PD1 inhibitors in the context of a pregnant cHL survivor could potentially lead to maternal and fetal complications as well as increase the risk of relapse. Pregnancy and fetal outcomes following PD1 inhibitors have been reported in women with melanoma, but not cHL. Such data may help physicians counsel their patients on this topic.

**Case:**

This case describes a 25‐year‐old woman who was diagnosed with advanced stage cHL that was treated with multiple courses of chemotherapy and autologous stem cell transplant (ASCT) for primary refractory disease. She experienced a relapse eight months following ASCT and was treated with the PD1 inhibitor pembrolizumab. She completed a total of 21 cycles, achieving a CR after cycle five. After 2 years of sustained CR off pembrolizumab, she had an unassisted and uneventful pregnancy. She delivered a healthy baby boy with no significant complications. He reached his normal milestones in his first year. She remains in CR four years following her last dose of pembrolizumab, evoking the possibility of her being cured of cHL.

**Conclusion:**

Successful pregnancies and fetal outcomes, while maintaining clinical remissions, are possible in women with relapsed cHL treated with pembrolizumab.

## 1 INTRODUCTION

Classical Hodgkin lymphoma (cHL) is one of the most common cancers diagnosed in adolescents and young adults (AYA), with an incidence of 4.2 per 100 000 in young adults in the United States.^1^ Advanced‐stage cHL is treated with curative intent using multi‐agent chemotherapy, either doxorubicin/bleomycin/vinblastine/dacarbazine (ABVD), brentuximab vedotin plus doxorubicin/vinblastine/dacarbazine (A + AVD), or bleomycin/etoposide/doxorubicin/ cyclophosphamide/vincristine/procarbazine/prednisone in escalated dose (BEACOPPesc)‐based therapy.^2^, ^3^ In the relapse setting, salvage high‐dose chemotherapy followed by autologous stem cell transplant (ASCT) remains the standard of care for eligible patients.^2^

New therapeutic options are available for relapsed cHL patients who are not eligible for ASCT or who have progressive disease following ASCT. Antibodies targeting the programmed cell death protein 1 (PD1) on T cells are effective in approximately 70% of patients with relapsed cHL.^4^, ^5^ The PD1 receptor, along with its ligand PD1‐ligand (PDL1), is part of an important immunosuppressive pathway, commonly referred to as an “immune checkpoint”. Binding of PDL1, expressed on tumor cells, to the PD1 receptor on T cells suppresses T cell effector function, favoring tumor growth, and evasion of immune surveillance.^6^ Recently, the KEYNOTE‐204 study demonstrated a significant progression‐free survival advantage for relapsed cHL patients treated with pembrolizumab, a PD1 inhibitor, over the anti‐CD30‐conjugated antibody brentuximab vedotin (BV).^7^ Given their success in the relapse setting, anti‐PD1 agents are currently being explored in the newly‐diagnosed setting^8^, ^9^; therefore, it is expected that more patients affected by HL will be exposed to PD1 inhibitors in the future and thus any information regarding their long‐term outcomes is essential.

Pregnancy is a particularly important issue in AYA cHL survivors. Patients in complete remission (CR), including those treated with ASCT, BV, or PD1 inhibitors, may wish to start families. Unfortunately, the data in the literature regarding pregnancy outcomes post‐PD1 inhibitors in cHL patients are scarce, making it difficult for treating physicians to counsel their patients on this topic. The PD1/PDL1 axis is essential in the maintenance of pregnancy^10^, ^11^; Patients who have become pregnant while on PD1 inhibitors for metastatic melanoma have experienced immune‐related and fetal complications, including intra‐uterine growth restriction (IUGR).^12^, ^13^

We are reporting the case of a successful pregnancy in a woman who achieved a CR with pembrolizumab given as a third‐line treatment for her relapsed cHL, effectively demonstrating that normal pregnancies and good fetal outcomes are possible in this population.

## 2 CASE

**FIGURE 1:**
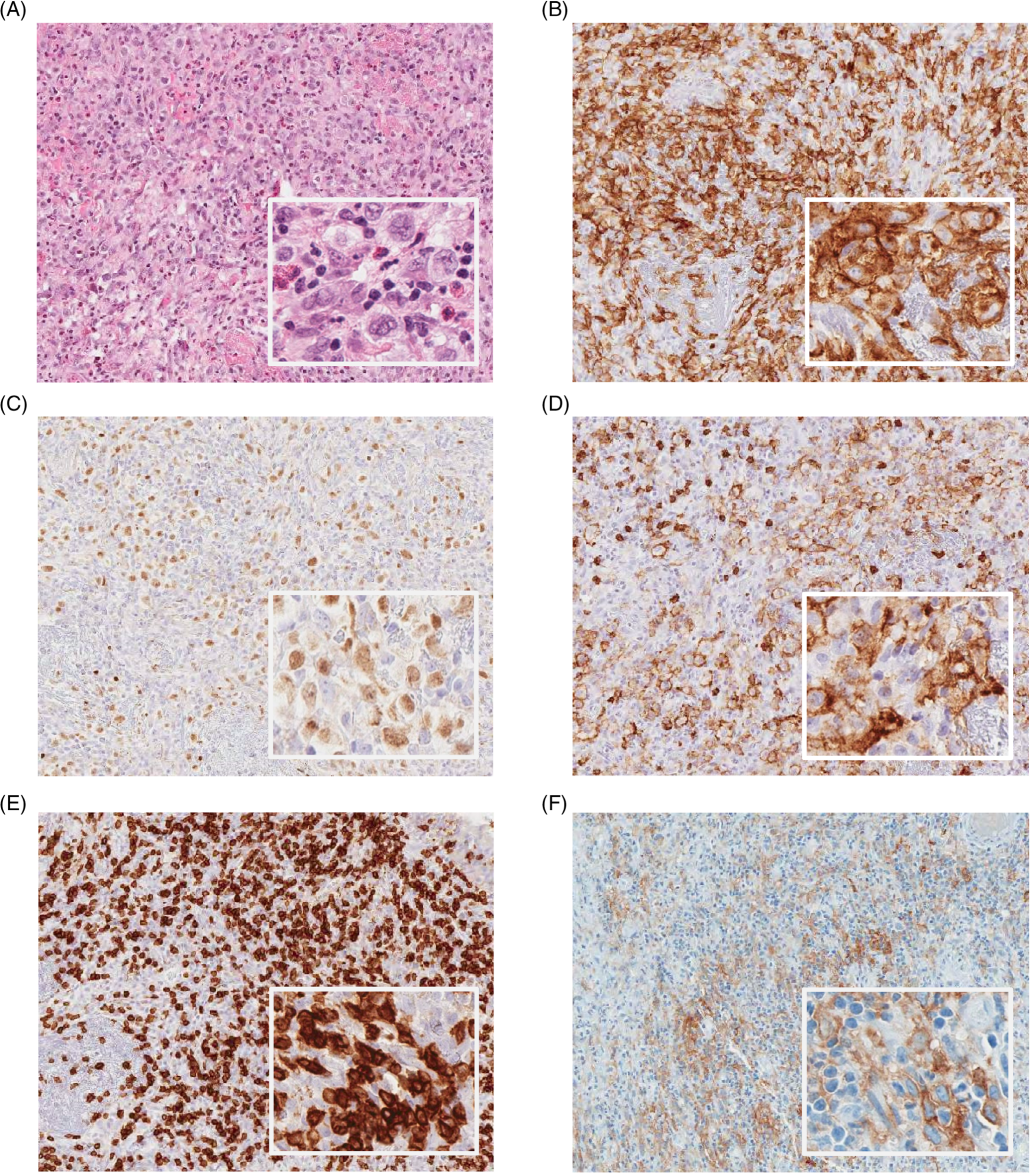
Initial biopsy: Immunostaining of the initial biopsy, comprised of lymphoid and lung tissue, obtained via Aperio scanner. Panels: (A) H&E (original magnification ×20 and ×100). (B) CD30 (tumor marker, original magnification ×20 and ×100). (C) PAX‐5 (transcription factor expressed in B‐cell maturation, original magnification ×20 and ×100). (D) CD20 (B‐cell antigen, original magnification ×20 and ×100). (E) CD3 (T‐cell co‐receptor, original magnification ×20 and ×100). (F) PDL1 (checkpoint inhibition ligand, original magnification ×20 and ×100). These immunohistochemical findings were most consistent with a diagnosis of cHL

**FIGURE 2:**
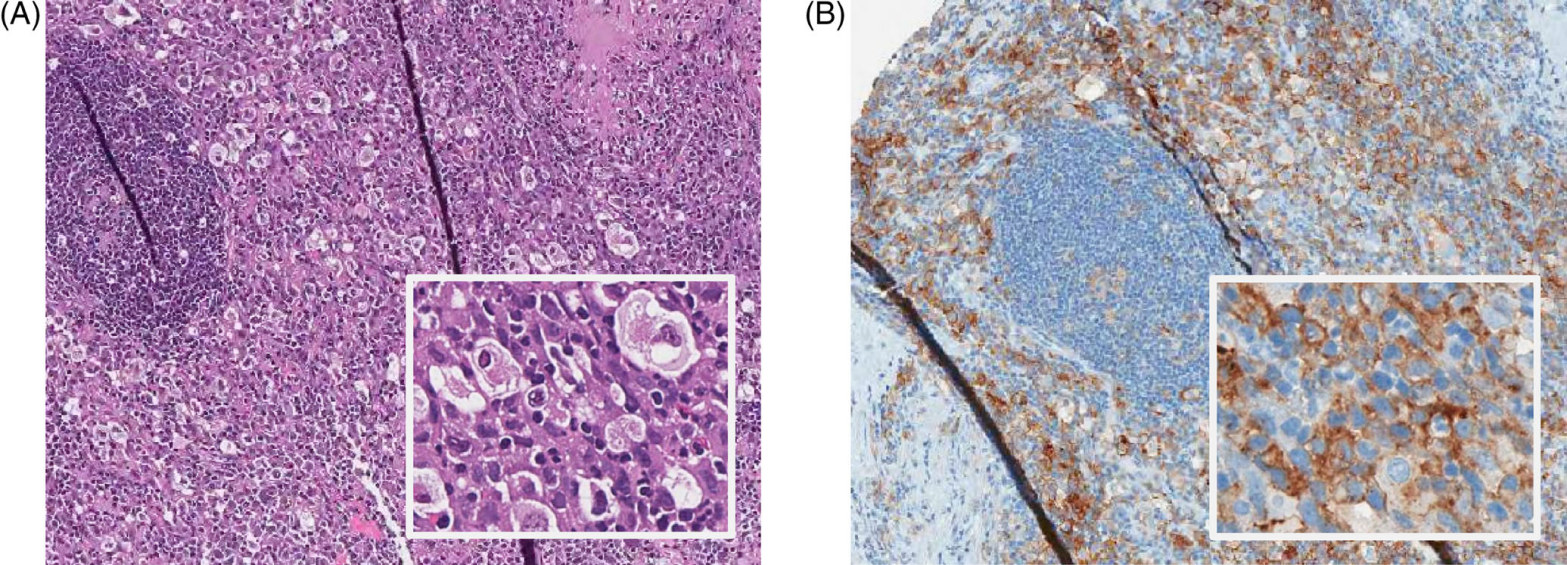
Relapse biopsy: Immunostaining of the relapse biopsy, comprised of lymphoid tissue, obtained via Aperio scanner. Panels: (A) H&E (original magnification ×20 and ×100). (B) PDL1 (checkpoint inhibition ligand, original magnification ×20 and ×100). These immunohistochemical findings were most consistent with a diagnosis of relapsed cHL

This previously healthy 25‐year‐old woman initially presented with a 2‐month history of cough, fever, night sweats, weight loss, and chest pain. On examination, she had an anterior chest wall mass protruding from the manubriosternal junction. Chest radiography revealed an enlarged right hilum adjacent to an anterior mediastinal mass. A biopsy of the mass (Figure 1, panels A‐E), comprising lung and lymphoid tissue, showed numerous mononuclear Hodgkin and occasional Reed‐Sternberg (HRS) cells. Overall, the findings (*refer to* *supplementary data 1*) were interpreted as most consistent with cHL. As numerous bones and both lungs were involved, advanced stage disease (IV) was diagnosed. The patient achieved a partial remission (PR) after 2 cycles of ABVD; however, following the sixth cycle, a Positron Emission Tomography (PET) scan demonstrated persistent fluorodeoxyglucose‐avid nodes in the mediastinum (Deauville 4^14^), for which she received a radical course of localized external beam radiotherapy to the mediastinum (45 Gy). A repeat PET scan performed a month after radiation confirmed a reduction in the size of the mediastinal mass, but recurrent lung nodules were noted at the original site of disease. These nodules were deemed too risky to biopsy and she was treated with three cycles of gemcitabine, dexamethasone, and cisplatin (GDP), achieved a PR and proceeded with ASCT in January 2015. A PET scan post‐ASCT was consistent with CR. However, eight months following ASCT, new cervical nodes and bone lesions appeared. A lymph node needle core biopsy (Figure 2, panels A‐B) showed numerous large atypical HRS cells in an inflammatory background, displaying a similar immunophenotype to the one observed in the initial biopsy (*refer to* *supplementary data 2*): these findings were consistent with relapsed cHL. She received radiation to a painful bone lesion at C7 and then enrolled in the KEYNOTE‐087 trial, a phase 2 trial assessing the efficacy and safety of pembrolizumab in relapsed cHL.^4^ The patient achieved a CR after 5 cycles and discontinued after 21 cycles in continued CR in November 2016, as the protocol allowed to cease therapy following two successive scans demonstrating CR.

After 2 years of sustained CR off pembrolizumab, that is, in November 2018, the patient requested counseling regarding pregnancy. There was some evidence supporting the safety of pregnancy to patients and their babies in lymphoma survivors who had previously been treated with traditional chemotherapy, but not PD1 inhibitors.^15^ Two theoretical risks were identified. First, there was a risk of IUGR given this has been reported in pregnant melanoma patients treated with immunotherapy. The second was the risk of relapse given that her sustained remission was only achievable through PD1 blockade: pregnancy‐induced suppression of the PD1/PDL1 axis could thus allow unabated growth of residual HRS cells.

Despite these risks, the patient became pregnant via natural conception a few months later. Her pregnancy was uneventful, with normal fetal growth and normal anatomic surveys being documented on routine ultrasounds. She eventually delivered a healthy newborn boy who weighed 3100 g, with normal APGARs of eight at 1 min, and nine at 5 min. The infant has been achieving his normal milestones as of one year of age. Repeat scans also demonstrate that the patient is still in CR, 4 years after her last dose of pembrolizumab.

## 3 DISCUSSION

This is the first reported case describing a successful pregnancy following treatment with a PD1 inhibitor in a patient with relapsed cHL, demonstrating that normal pregnancies and good fetal outcomes are possible in these patients. In animal models, the PD1/PDL1 axis has been shown to be vital in the maintenance of pregnancy through the induction of maternal tolerance to fetal tissue, in part through up‐regulation of PDL1 in trophoblasts and regulatory T cells at the utero‐placental interface.^10^ In fact, PD1 inhibitors have caused spontaneous abortions in animals and are considered to pose a risk to human fetuses, and are thus labeled Category D by the Food and Drug Administration.^11^, ^16^ Nonetheless, several patients with metastatic melanoma have had successful pregnancies on PD1 inhibitors, with or without inhibitors to CTLA‐4.^12^ Some of these cases have been associated with both maternal and fetal immune‐related adverse events (irAE), including IUGR in two infants.^12^, ^13^, ^17^, ^18^ However, upon delivery, these babies and infants achieved normal developmental milestones. It is noteworthy that the patients in these case reports were taking their immune checkpoint inhibitors during their pregnancy, and that these drugs are IgG4 antibodies that can cross the placental barrier.^16^ Therefore, that no irAE or IUGR were observed in our patient may simply reflect the fact that pembrolizumab was no longer present in her body, as her last dose was two years prior to conception.

Given that the PD1/PDL1 axis is used as a mechanism of immune escape by HRS cells, there was a concern for a cHL relapse during this patient's pregnancy. The HRS cells in this patient expressed high levels of PDL1 (Figure 1, panel F; and, 2, panel B), a biomarker that has been associated with amplification of the *PDL1* gene locus, poor response to ABVD, and favorable responses to PD1 blockade.^19^ Thus, there was a theoretical risk that PD1‐mediated suppression of T cell function, as well as other immune suppressive effects observed in pregnant women, would favor tumor growth.^20^, ^21^ Several retrospective studies have shown that pregnancy is not associated with an increased risk of relapse in HL survivors after treatment with first‐line chemotherapy, most commonly ABVD.^15^, ^22^ However, most of the pregnancies occurred beyond 2 years of primary therapy, when the risk of relapse would have been very low and thus most of these women would have been cured. Another potential selection bias is that they included only patients who have achieved a CR and had not experienced a relapse within 6 months of finishing primary therapy, thus selecting women with lower‐risk disease that were more likely to be cured and subsequently decided to become pregnant.^15^ The effect of pregnancy on the risk of a subsequent relapse in patients who have already experienced a prior relapse is not clear and has never been reported in anti‐PD1‐treated cHL patients.

With increased utilization of PD1 inhibitors in young patients with relapsed cHL and improvements in survival in this population, we will likely observe a growing number of patients who will seek counseling regarding pregnancy. Although this is a single case, it highlights that pregnancy and good fetal outcomes are possible in female cHL survivors who have achieved CRs with PD1 inhibitors. There are not enough data to suggest that pregnancy will not increase the risk of relapse in high‐risk patients, especially if the duration of CR is short. However, the fact that relapse has not occurred in this patient, four years after stopping pembrolizumab, even after the immunological stress of pregnancy, evokes the possibility that similarly to BV,^23^ a proportion of patients with relapsed cHL may be cured with PD1 inhibitors.

## CONFLICT OF INTEREST

NAJ has received consultant fees from Merck and Bristol Myers Squibb. ALN, RNR, and TPH have no conflicts of interest to disclose.

## AUTHOR CONTRIBUTIONS

**Alexandre Le‐Nguyen:** Writing‐original draft; writing‐review & editing. **Ryan Rys:** Visualization; writing‐review & editing. **Tina Petrogiannis‐Haliotis:** Visualization; writing‐review & editing. **Nathalie Johnson:** Conceptualization; funding acquisition; supervision; writing‐original draft; writing‐review & editing.

## ETHICS STATEMENT

This project was approved by the Research Ethics Board (18‐030). Informed consent from the patient was obtained for publication of this report.

## Supporting information


**Appendix S1.** Supporting Information

## Data Availability

The data that support the findings of this study are available from the corresponding author upon reasonable request.
