# Supplementary material for: Successful pregnancy and fetal outcome following previous treatment with pembrolizumab for relapsed Hodgkin's lymphoma
Source: Cancer Rep (Hoboken). 2021 May 28;5(1):e1432. doi: 10.1002/cnr2.1432 (PMC8789614; doi:10.1002/cnr2.1432)
Supplement: Supplementary file 1 — Appendix S1. Supporting Information [file CNR2-5-e1432-s001.docx]

**Supplementary data 1**

A biopsy of the mass (Figure 1, panels A-E), comprising lung and lymphoid tissue, showed subtotal effacement of tissue architecture by a frequently necrotic atypical polymorphous infiltrate that exhibited numerous mononuclear Hodgkin and occasional Reed-Sternberg (HRS) cells, frequently clustered, in an inflammatory background that mainly comprised granulocytes (neutrophils and eosinophils), lymphocytes (including marked T-cells, CD4>>CD8), histiocytes and plasma cells. The large atypical HRS cells were positive for CD30, CD15, MUM-1, PAX-5 (weak); OCT2 (weak), BOB.1 (weak) and CD20 (weak to moderate). They were negative for CD45, CD43, TIA-1, CD3, CD4, CD8, EMA, ALK-1, TdT, CD79a, CD10, CD23 and EBER. Overall, the findings were interpreted as most consistent with cHL, difficult to subclassify in this specimen and showing a greater than usually seen preservation of B-cell markers (including CD20 and BOB.1).

**Supplementary data 2**

A lymph node needle core biopsy (Figure 2, panels A-B) showed an atypical polymorphous infiltrate, exhibiting numerous large atypical (HRS) cells (including a number of lacunar and a rare mummified cell), in an inflammatory background that comprised lymphocytes, histiocytes, plasma cells and polymorphonuclear cells (including abundant eosinophils). The atypical infiltrate was characterized by the presence of paucicellular collagen bands, partly delineating atypical nodules. Immunohistochemical studies showed the HRS cells displaying a similar immunophenotype to that observed in the initial biopsy but absent CD20 (not shown).
